# Supplementary figures and images for: Kunitz type protease inhibitor EgKI-1 from the canine tapeworm Echinococcus granulosus as a promising therapeutic against breast cancer
Source: PLoS One. 2018 Aug 31;13(8):e0200433. doi: 10.1371/journal.pone.0200433 (PMC6118354; doi:10.1371/journal.pone.0200433)

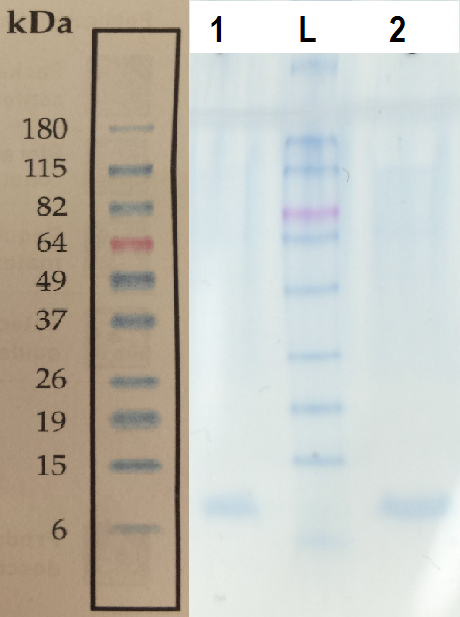

Supplement: S1 Fig — 1, 3 μg EgKI-1; L, molecular size markers; 2, 6 μg EgKI-1. (TIF) [file pone.0200433.s001.tif]

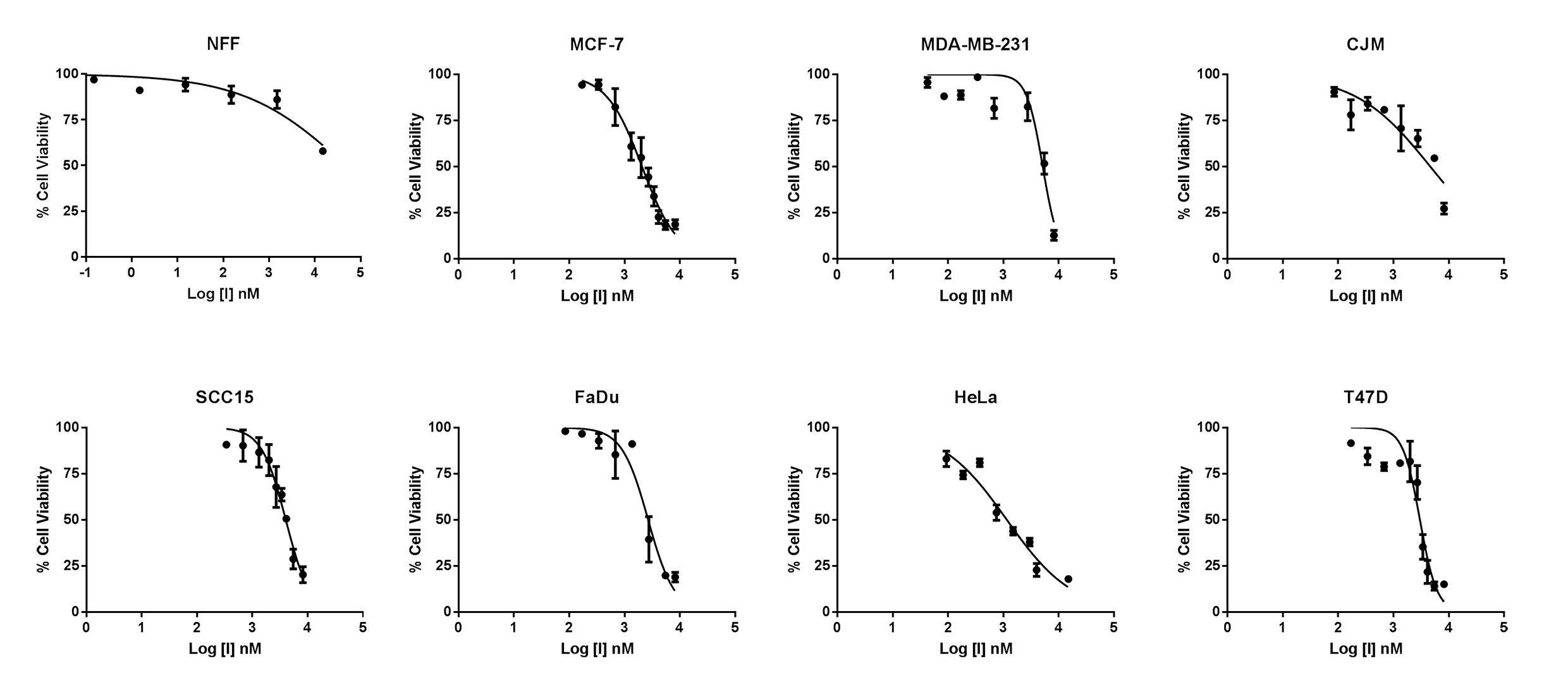

Supplement: S2 Fig — Viable cell percentages of primary neonatal foreskin fibroblast cells (NFF), breast cancer cell lines (MCF-7, MDA-MB-231, T47D), melanoma (CJM), squamous cell carcinomas (SCC15, FaDu) and cervical adenocarcinoma (HeLa) cell lines with different EgKI-1 concentrations in vitro. (TIF) [file pone.0200433.s002.tif]

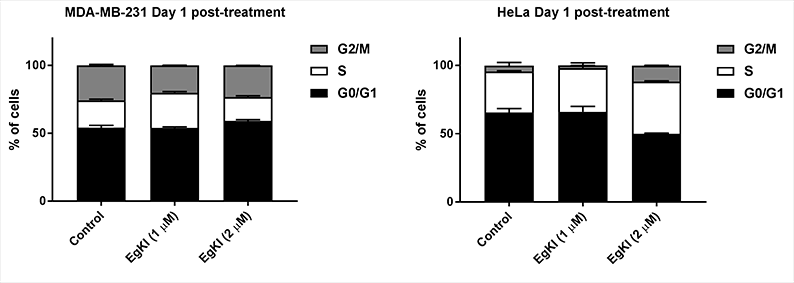

Supplement: S3 Fig — MDA-MB-231 and HeLa cells were treated with 1 μM and 2 μM EgKI-1 for 24 hours and the cell cycle distribution was determined by flow cytometry. No significant difference was observed among the different cell cycle phases. (TIF) [file pone.0200433.s003.tif]

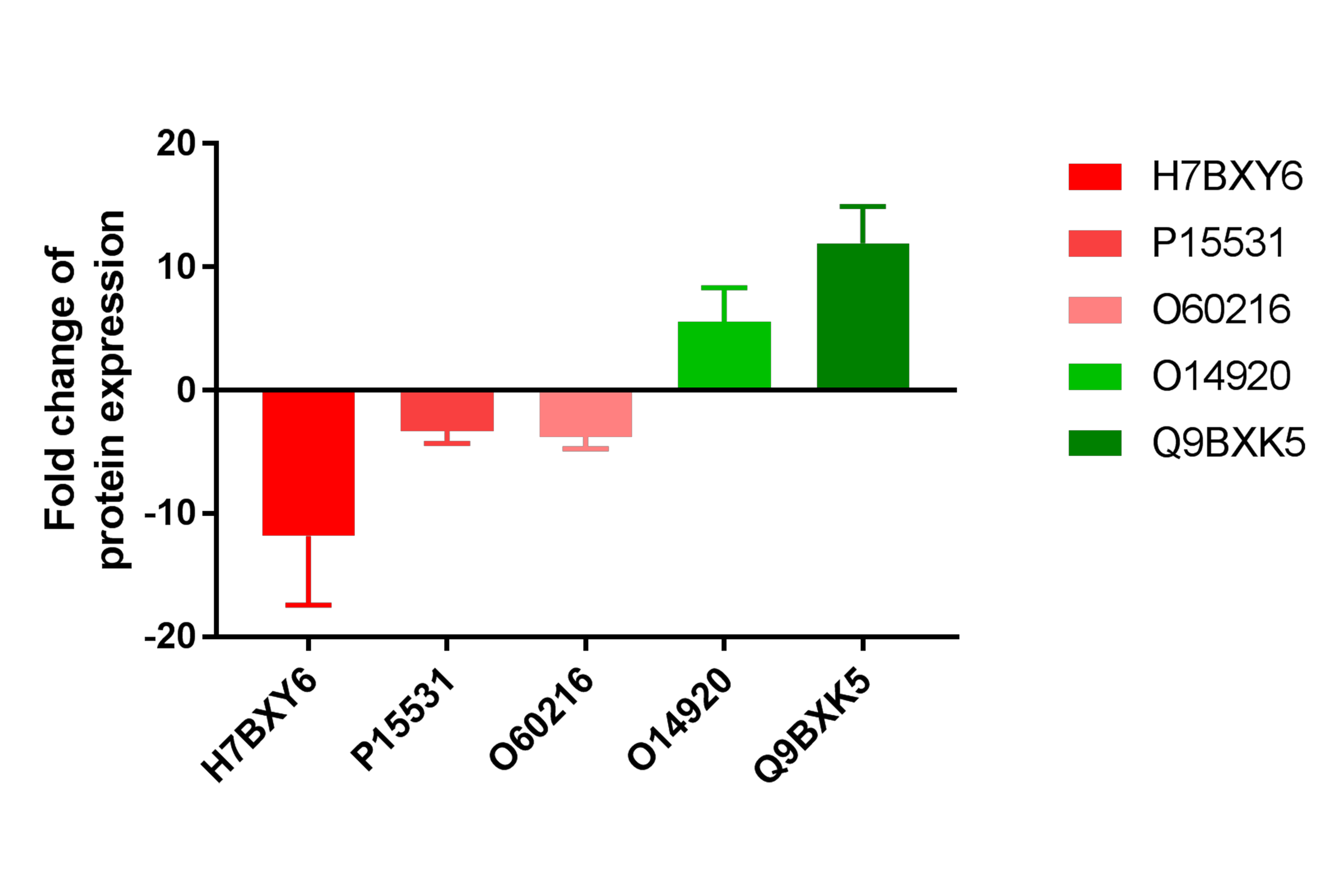

Supplement: S5 Fig — H7BXY6:Tetraspanin, P15531:nucleoside diphosphate kinase A, O60216:double-strand-break repair protein, O14920:inhibitor of nuclear factor kappa-B kinase subunit beta, Q9BXK5:Bcl-2-like protein 13. (TIF) [file pone.0200433.s005.tif]

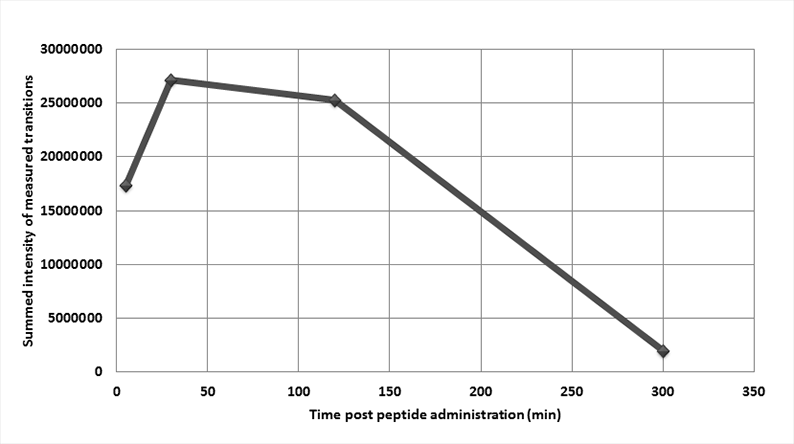

Supplement: S6 Fig — After intraperitoneal injection into mice, EgKI-1 was absorbed into the blood within 5 minutes and had been cleared in 5 hours. (TIF) [file pone.0200433.s006.tif]
