# Supplementary material for: Kunitz type protease inhibitor EgKI-1 from the canine tapeworm Echinococcus granulosus as a promising therapeutic against breast cancer
Source: PLoS One. 2018 Aug 31;13(8):e0200433. doi: 10.1371/journal.pone.0200433 (PMC6118354; doi:10.1371/journal.pone.0200433)
Supplement: S4 Fig — Heat map analysis of 1770 proteins among three biological replicates of the control and EgKI-1 treated samples after 30 min, 2 hours, 4 hours and 24 hours. The fold change value of the MS signal intensity is shown. (PDF) [file pone.0200433.s004.pdf]

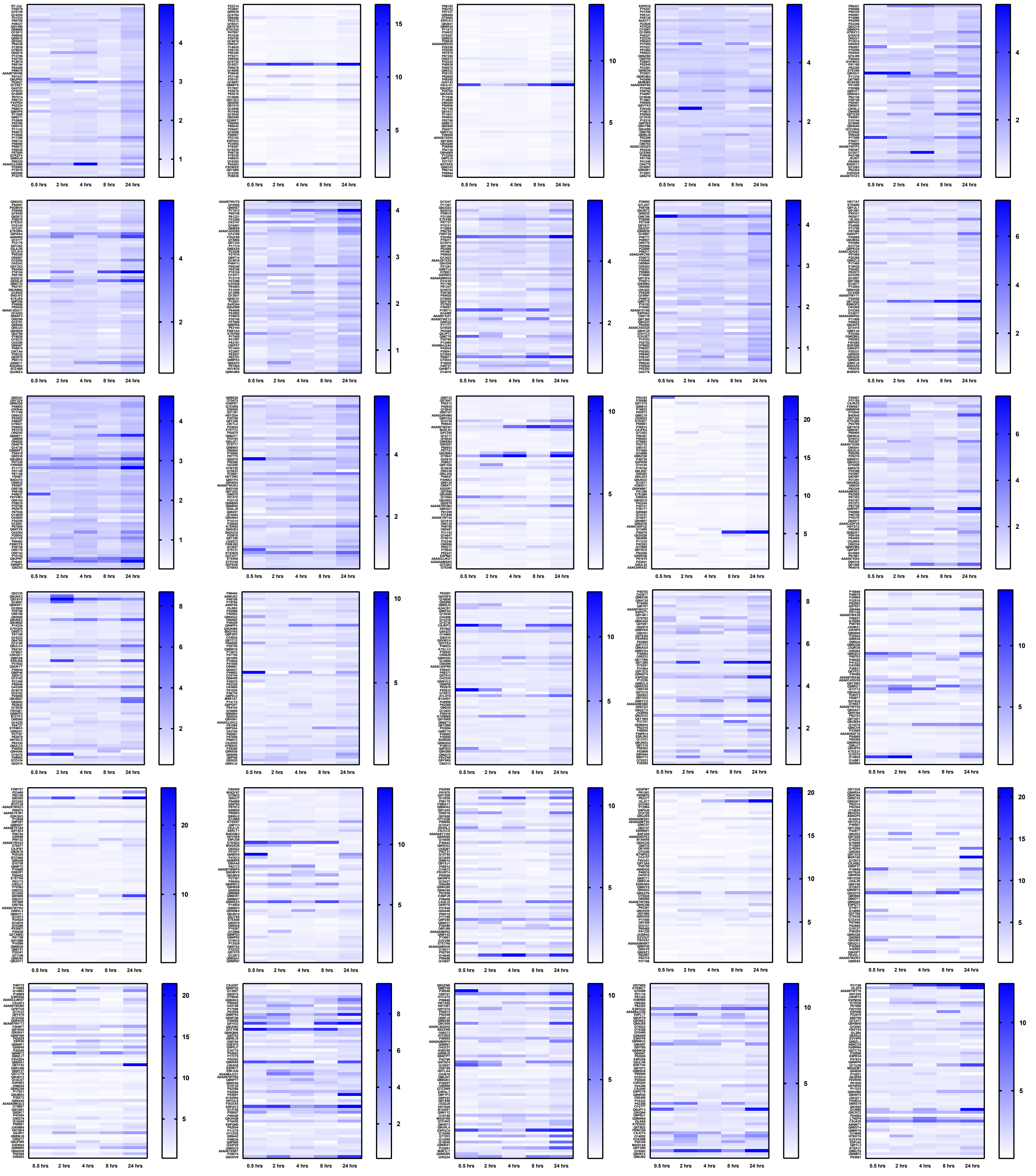


Supplementary Figure S4
